# Melanin nanoparticles as a novel contrast agent for optoacoustic tomography

**DOI:** 10.1016/j.pacs.2015.02.001

**Published:** 2015-02-14

**Authors:** Anton Liopo, Richard Su, Alexander A. Oraevsky

**Affiliations:** TomoWave Laboratories, Houston Texas 77081, United States

**Keywords:** Optoacoustic, photoacoustic, gold nanorod nanoparticles, melanin nanoparticles, image contrast

## Abstract

We describe the synthesis and characterization of melanin-like nanoparticles (MNP) as novel contrast agents for optoacoustic tomography. Good dispersion stability of high concentration MNPs in different biological media was achieved with thiol-terminated methoxy-poly(ethylene glycol), which can be used for further functional conjugation. MNP-PEG were found biocompatible with human MCF-7 and 3T3 cells. Cell toxicity of MNPs was found lower than that of gold nanorods for concentrations that provide equal optical absorbance. Optoacoustic tomography images were obtained with Laser Optoacoustic Imaging System (LOIS-3D) from tubes filled with contrast agents and live mice. Imaging of tubes permitted verification of the system resolution <300 μm and sensitivity Δμa=0.03/cm under safe laser fluence of 20 mJ/cm^2^. Water suspensions of MNP demonstrated optoacoustic efficiency that is about equal to that of gold nanorods under conditions of equal optical absorption. We conclude that MNPs have the potential for biomedical imaging applications as optoacoustic contrast agents.

## 1 Introduction

Optoacoustic tomography (OAT) is a biomedical imaging modality that combines the spectral selectivity and the high optical contrast based on variation of optical absorption with the high resolution based on detection of ultrasound generated in tissues with nanosecond laser pulses [1,2]. Typically, lasers emitting in the near-infrared (NIR) spectral range from ∼700 nm to ∼1300 nm are used for generation of OA signals (and images) due to the relatively weak absorption of biological tissues in this spectral range, also known as the window of optical tissue transparency [3]. Even though OAT resolution is scalable with approximate ratio of depth of imaging to resolution of about 200 [4], the most significant value of this technology is expected from its capability of visualizing deep tissue structures [5–8] and potentially providing high contrast, high resolution quantitative volumetric information about molecular content of biological tissues.

Hemoglobin and oxy-hemoglobin of blood are the main tissue chromophores in the NIR spectral range [9], therefore OAT may be naturally defined as a functional imaging modality for characterization of blood distribution in the live body. On the other hand, not many molecules of biomedical interest possess strong optical absorption in the range of the optical tissue transparency. Therefore, application of contrast agents (CA) that target non-absorbing molecules and cells is important for molecular optoacoustic imaging. A large number of optical and optoacoustic CA has been developed since early 2000s, and many of them found applications in preclinical research using live animal models [10]. The signal amplitude emitted by a contrast agent is proportional to its volume accumulated at the target site. Therefore, nanoparticles, such as gold nanorods, having their volume and the optical absorption coefficient much larger than those of any molecular probe, are thought to be of especially significant value as contrast agents for optoacoustic imaging [11]. All nanoparticle based optoacoustic CA can be divided in two groups: nanoparticles based on exogenous or endogenous chromophores. While CA development in the previous decade was focused on exogenous nanoparticles, recently the deserved attention is gradually shifting towards less toxic nanoparticles based on endogenous molecules [12]. Two of the biggest advantages of using endogenous contrast agents for imaging applications are safety and the possibility of revealing the true physiological conditions, because the physiological parameters are not altered during optoacoustic image acquisition.

### 1.1 Optoacoustic contrast opportunity offered by Melanin

Melanin molecules have much stronger optical absorption than surrounding skin in the near-infrared region [13]. However, unlike hemoglobin and oxy-hemoglobin, imaging of melanin in skin is not used for biomedical diagnostics, with exception of assessment of skin protection from ultraviolet radiation. We would like to employ strong NIR absorption of melanin to enhance optoacoustic contrast of tissues, such as cancerous tumors, which can potentially accumulate significant concentration of melanin nanoparticles. The broad optical absorption spectrum of melanin makes it suitable for optoacoustic imaging with any available laser wavelength [14]. Melanins (may be surprisingly) are widely distributed in many parts of living organisms. Melanins are involved in various functions, including photosensitization, metal ion chelation, thermoregulation, protection from radiation and free radical quenching, a vital property in the regulation of oxidative stress [15,16]. Melanins are usually categorized into two major types according to the difference in precursors and colors: brown-black eumelanins and yellow-red pheomelanins [17]. Unlike fluorescent proteins, melanin cannot be used for studies of subcellular protein distribution and interaction analysis, but it has the advantage of being visible by such noninvasive deep tissue imaging as optoacoustics. An advantage of melanin compared with fluorescent proteins is its very good stability in physiological environment of live animals [18].

Previously, optoacoustic imaging has been used to detect melanin in lymph node metastases from melanoma cancer. Differentiation between blood and melanoma proved to be difficult because both are strong optical absorbers and therefore create comparable optoacoustic signals [19]. OAT was previously proposed for diagnosis, prognosis, and treatment planning of melanotic melanoma (>90% of all melanomas) [20]. Several groups successfully demonstrated gene delivery technique for overexpression of melanin in cells loaded with tyrosinase, which resulted in melanin contrast for optoacoustic (photoacoustic) microscopy [21,22]. Melanin was also shown to be a suitable target for laser-induced thermotherapy [18]. The tyrosinase gene can be utilized as a multifunctional reporter gene for optoacoustic, magnetic resonance and positron emission tomography *in vitro* and *in vivo* [23]. On the other hand, the process of melanin production in transfected cells is quite toxic [24]. For example, epidermal melanocytes are particularly vulnerable to oxidative stress owing to the pro-oxidant state generated during melanin synthesis, and to the intrinsic antioxidant defenses that are compromised in pathologic conditions. Melanin synthesis involves oxidation reactions and superoxide anion (O_2_^−^) and hydrogen peroxide (H_2_O_2_) generation, which subject melanocytes to oxidative stress [25]. Therefore, we support promising application of melanin as a contrast agent formulated as nanoparticles. Melanin is an effective scavenger of free radical toxicity. Application of melanin-based nanoparticles has been demonstrated as a protective agent against GNR induced neurotoxicity in mice [26], against ionizing radiation [27] and, thus, MNP may be used as a contrast and protection nanoplatform for different imaging modalities [28]. There are usually two approaches toward fabrication of melanin nanoparticles: nanoparticle formation from melanin isolated from natural sources and synthesis of an artificial MNPs. Natural melanins have been obtained by separation and purification of the pigment from their biological environment and these procedures need to be developed to obtain the unmodified characteristics of natural melanins [29]. Synthetic melanin models are usually prepared by chemical oxidation of dopamine [30] or enzymatic oxidation of precursor molecules such as tyrosine and 3,4-dihydroxy-L-phenylalanine. While physical and chemical properties of melanin are preserved in the process of fabrication, synthetic melanin models usually could not provide the particle shape and were insoluble in water [31]. However, in the past several years there have been developments in synthetic methods to prepare size-controllable melanin-like nanoparticles having a good dispersibility in water and biological media [29,32,33]. High dispersibility and dispersion stability of nanoparticles is critically important for two aspects of *in vivo* applications. The first, administration of the contrast agents has to be made in significantly enhanced concentrations in order to make their optical absorbance competitive with red blood cells. The second aspect is that effective PEGylation of nanoparticles needed for high MNP dispersibility in biological media simultaneously make these nanoparticles invisible to reticulo-endothelial system [34].

Our report is focused on three MNP-related aspects: (i) the dispersion stability of MNP-PEG conjugates, (ii) the toxicity of PEG-MNP conjugates in different cell cultures and *in vivo* and (iii) the investigation of MNP as a contrast agent for optoacoustic imaging.

## 2 Materials and methods

### 2.1 Reagents

The chemicals were obtained at the highest purity available and used as received from commercial sources: dopamine hydrochloride (Sigma Aldrich), sodium hydroxide (NaOH, Sigma), hexadecyltrimethylammonium bromide (CTAB, Sigma), ammonia hydroxide (NH_4_OH, Sigma-Aldrich), potassium carbonate (K_2_CO_3_, Sigma-Aldrich), poly (ethylene glycol) methyl either thiol or methoxypolyethylene glycol thiol mPEG thiol, MW 5000, (mPEG-Thiol or PEG, Laysan Bio Inc.), gold(III) chloride trihydrate or chloroauric acid trihydrate (HAuCl_4_·3H_2_O, Aldrich), sodium borohydride (NaBH_4_, Aldrich), silver nitrate (AgNO_3_, Sigma- Aldrich). Ultrapure water (18.2 MΩ·cm at 25 °C) was used throughout the work.

### 2.2 Synthesis of Water Dispersible MNPs.

Water-dispersible MNP were prepared according to the protocol described originally described in [29] by an oxidation and polymerization of 3,4-dihydroxy-phenylalanin (DOPA) with KMnO_4_ [32]. A total of 50 mg dopamine hydrochloride was dissolved in 20 mL of deionized water. Under vigorous stirring, 40 to 400 μL of 1 N NaOH was added to a dopamine hydrochloride solution at 60 °C. Instead of originally proposed 4 hours [29], we kept the reaction overnight at pH=10 and achieved a more homogeneous distribution of MNPs. The experiments were conducted with 200 μL of sodium hydroxide. The color of the solution turned to pale yellow as soon as NaOH was added and gradually changed from transparent light to very dark brown. After reacting overnight, MNPs were retrieved by dual centrifugation. In contrast to original single centrifugation, we first used low-speed centrifugation (2500 g, 10 min) and collected supernatant discarding pellet of heavy large-sized aggregated materials. Then we performed a high-speed centrifugation (16000 g, 20 min, RT), collected the pellet and washed it twice with deionized water. To increase the working concentration of the MNP solution, high speed centrifugation (16000 g, 20 min) could be repeated.

### 2.3 Surface Modification of MNP by PEGylation.

For optimization of PEGylation of MNP, using our previous experience with gold nanorods (GNR) [34], we modified the PEGylation method previously reported in [33]. To achieve a better PEGylation, 1.0 mL of 2 mM potassium carbonate (K_2_CO_3_) was added to 8 mL of aqueous MNP solution (0.5 mg/mL of water), and 1.0 mL of mPEG-Thiol-5000 (molecular weight 5000, Laysan Bio Inc.) was added in concentration 10 mM (i.e. C=5.0 mg/mL). NH_4_OH solution (28 wt %) was added to adjust the pH to between 9 and 10 to stabilize the reactive medium [29]. In accord with our previous studies [34], in the final stage of PEGylation we added K_2_CO_3_ to activate SH group of mPEG-Thiol molecule in order to achieve better binding to the surface of the nanoparticle. After rigorous stirring for 4 h (RT), surface-modified MNPs were obtained at two cycles of centrifugation – washing. Centrifugation was done at 16000 g for 15-20 min. The pellet was re-suspended in phosphate buffer solution (PBS) with neutral pH=7.4.

TEM images of MNP were obtained with high contrast transmission electron microscope JEOL 1230. In order to measure spectral properties of nanoparticles in the spectral range of 400 – 1100 nm we used UV-VIS-NIR Spectrophotometer Evolution-201 (Thermo Fisher Scientific, New Hampshire).

### 2.4 Gold Nanorods (GNR) as reference nanoparticles

The general strategy for the synthesis and stabilization of GNRs with thiol-terminal polyethylene glycol (mPEG-thiol) was adapted from our previously reported methodologies, where we used the displacement of the original bilayer of surfactant CTAB to provide biocompatibility of the resulting optoacoustic contrast agent with a narrow-band of optical absorption in the near-infrared spectral range [34,35].

### 2.5 In vitro cyto-toxicity, cell viability and cell proliferation tests

Two human cells lines were used for cyto-toxicity, cell viability and cell proliferation. These were MCF-7 (Human breast adenocarcinoma), and 3T3 cells (mouse embryonic fibroblasts). Cell lines were purchased from American Type Culture Collection (ATCC, Manassas, VA, USA) and were cultured in essential media with 10% fetal bovine serum (Hyclone) at 37.0 °C, 95% air and 5% carbon dioxide, with renewal of the medium every 2-3 days.

Cell toxicity and cell viability were measured using the LDH and MTT kits according to the procedure in the manufacturer's manual [36]. MCF-7 cells were seeded into a 96-well plate at a density of 10^5^ cells/ml in 0.125 ml of media per well. 3T3 cells were seeded in same plate, but at density of 5 × 10^4^ cells/ml. After 24 h incubation with gradually increasing concentrations of PEGylated MNP, 25 μL samples of the media were collected at indicated arch independence points in the LDH assay kit. The experimental condition was measured using the maximum amount of releasable LDH enzyme activity (Total or High LDH), which was determined by lysis of the cells in the medium with 1% Triton X-100 (at this concentration, Triton X-100 does not affect the LDH activity).

Cytotoxicity (%) was calculated as the ratio between LDH release from cells to medium (LDH_R_) to the total level of LDH_T_ after Triton X100 application plus LDH_R_ for each experimental condition of PEG-MNP incubation [34].

The MTT assay is a colorimetric assay for assessing cell viability. NAD(P)H-dependent cellular oxidoreductase is capable of reducing the tetrazolium dye 3-(4.5 dimethylthiazol 2-yl)-2,5-diphenyltetrazolium bromide (MTT) to its insoluble (E,Z)-5-(4.5 dimethylthiazol 2-yl)-1,3-diphenylformazan (Formazan), which has a purple color.

The absorbance of each well was measured with a necessary reference by using a microplate reader ELx800TM (Bio-Tek Instruments, VT), as described in the manuals for LDH and MTT assays. Each experiment was performed minimum in triplicate. As control, we used cells to which only PBS solution (pH 7.4) was added and as a background control we used media only.

### 2.6 Optoacoustic imaging system

**Figure 1:**
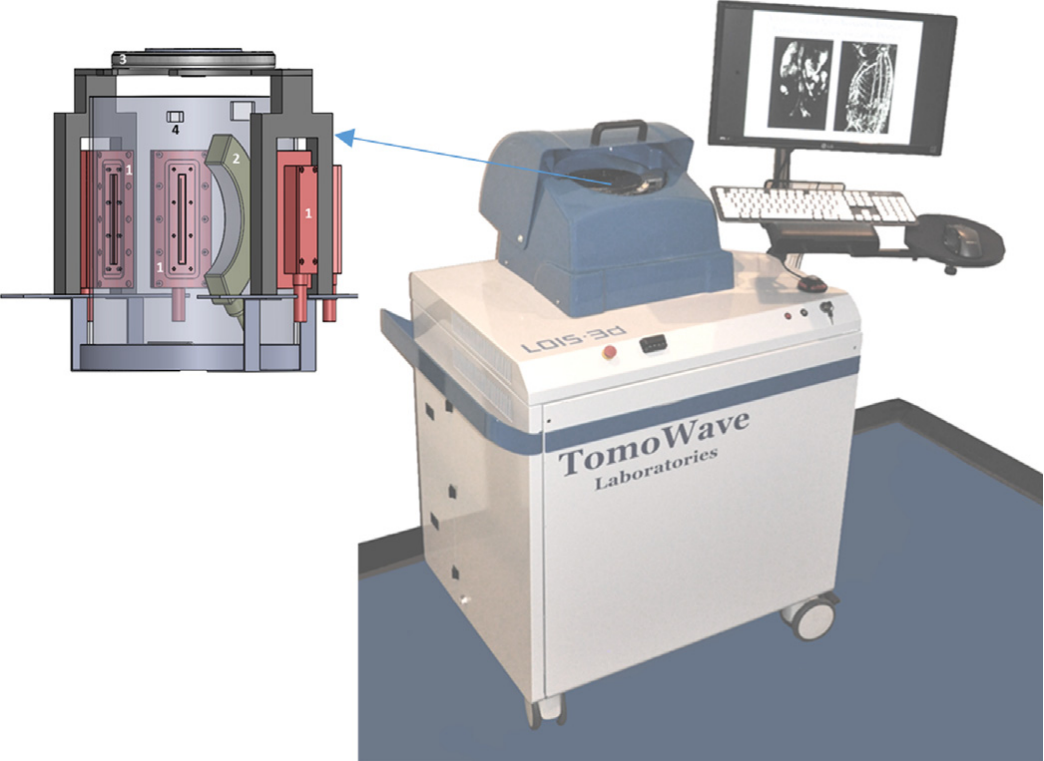
A photograph of three dimensional laser optoacoustic imaging system with detailed rendering of the imaging module, which include: 1 – quad fiber optic bundle of the light delivery system, 2 – arc array of ultrasonic transducers, 3 – computer controlled rotational stage, 4 – video camera.

Depicted in Figure 1 is the Laser Optoacoustic Imaging System, LOIS-3D (TomoWave Systems, Houston, TX), a commercial device for optoacoustic tomography research in small animals, was developed based on the laboratory prototype of TomoWave Laboratories, described in our earlier publications [6,37–39]. A q-switched Nd:YAG pumped Ti:Sapphire laser (SpectraWave-1x, a collaborative product of TomoWave, Houston, Texas and Quanta Systems, Solbiate Olona, Italy) provides 8 ns pulses with energy of 50 to 100 mJ wavelengths tunable from 750 nm to 850 nm as well as 1064 nm. For these experiments, the laser was set to 800 nm with a repetition rate of 10 Hz. The laser pulses were delivered to the object of imaging via double-bifurcated fiber bundle with 50% losses from its circular input to rectangular outputs placed on the surface of the imaging module at 45 and 90 degrees with respect to the detector array of 96 ultrasonic transducers (see Figure 1). This illumination geometry permitted close to homogeneous optical energy distribution through the object and imaging free of skin related artifacts. The arc shaped array of ultrawide-band ultrasonic transducers (bandwidth of 50 kHz to 8 MHz at -6 dB) provided an aperture of 115 deg with a radius of 65 mm, which resulted in spatial resolution better than 0.3 mm over the volume of 40 mm sphere. The optoacoustic coupling medium during scans was a degassed distilled water held with a thermostat at a constant temperature of 33 °C. Optoacoustic images were acquired by rotating the object 360 deg with steps of 1.2 deg using computer controlled rotational stage. The detected signals were digitized with a sampling rate of 40 MHz. Signal processing was similar to that described in our previous publications using deconvolution of transducer impulse response and a band pass filter [40]. Image reconstruction was made with filtered back projection algorithm utilizing half-time data set [41]. Three-dimensional visualization was done with TomoView, software package developed at TomoWave based on the open SDK source developed by Kitware (Clifton Park, NY). The image processing parameters such as scalar opacity, color mapping, and gradient opacity were held constant between all objects and scans such that one can directly compare the image brightness across all images in a quantitative manner and assign their colors using a linear color palette. From lowest to highest optoacoustic contrast is black to red with voxels deemed to be noise colored black (transparent).

### 2.7 Experiments in Phantoms

Because of their strong optical absorption resonance in the near infrared and spectral tenability based on aspect ratio, GNRs have become the gold standard for optoacoustic contrast agents [42,43]. Therefore, optoacoustic contrast of MNPs was compared with that of GNRs. A custom-made bracket supported two silicone tubes with internal diameter, internal diameter, ID=510 μm and wall thickness of 150 μm. One tube was filled with GNR nanoparticle suspension and the other was filled with MNP suspension in water. To separate GNR tube from MNP tube on optoacoustic images, the former had one knot and the latter had two knots. This phantom was used to perform a series of quantitative imaging experiments with LOIS-3D using pulsed laser energy of about 6.25 mJ out of each of the four fiberoptic illuminators, which corresponded to the total effective optical fluence of 2.5 mJ/cm^2^. Imaging experiments were repeated three times. The average brightness of optoacoustic images was measured in the central area of the images illuminated more evenly than the adjacent areas. The measurement of brightness in the tubes was done using the system's segmentation software. Each tube was identified and their mean brightness and standard deviations were calculated based on their image brightness values from the central area of the images, i.e. in the knot area of GNR tube and in the straight portion of the MNP tube between the knots.

### 2.8 Experiments with a live mouse

To test toxicity of MNP *in vivo* we used an Athymic Nude-Foxn1^nu^ mouse (Harlan, Indianapolis, Indiana), 8 weeks old, weighing about 29 g. Animal handling, isoflurane anesthesia, and euthanasia were described in detail in our previous publications [37–40]. All mouse related procedures were in compliance with our Institutional Animal Care and Use Committee (IACUC) protocol. The mouse was illuminated with 4 laser beams each providing the effective optical fluence on the skin of ∼0.63 mJ/cm^2^. The mouse was scanned prior to injection of MNP solution to provide the control optoacoustic images. After the control scan, the mouse was taken out of the imaging module and had 0.2 mL of the melanin nanoparticles suspension in PBS with concentration of about 30 mg MNP/mL injected intravenously through a tail vein. The mouse was placed back in its cage after the imaging experiment and received normal diet and water.

## 3 Results and Discussion

### 3.1 Synthesis and PEGylation of Melanin nanoparticles

**Figure 2:**
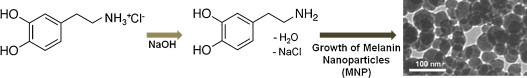
Scheme of the protocol for synthesis of melanin-like nanoparticles from dopamine hydrochloride.

**Figure 3:**
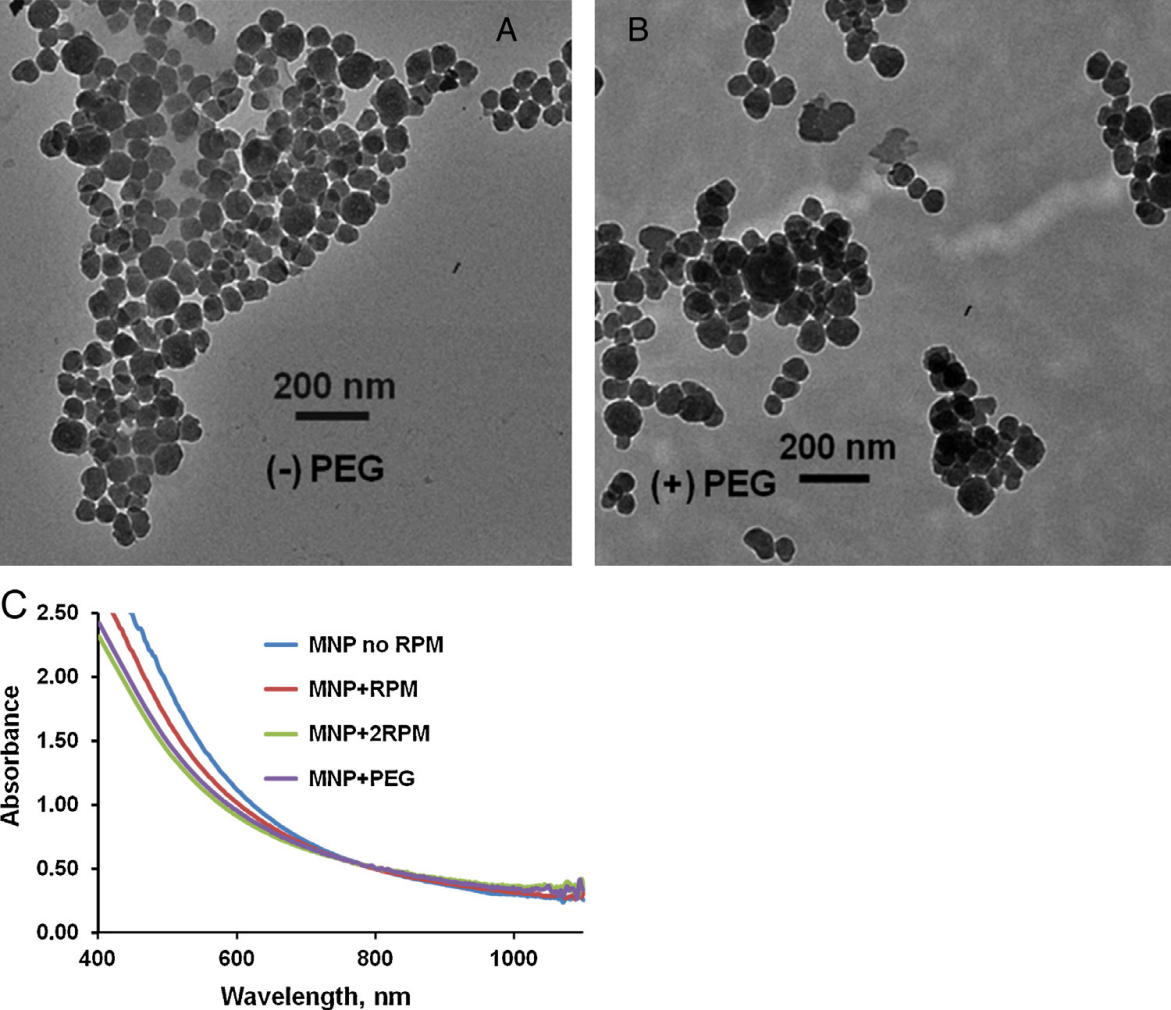
TEM images of melanin nanoparticles before (a) and after surface modification (b) with methoxy-poly(ethylene glycol)-thiol. (c) Optical absorption spectra of MNP after synthesis without high speed centrifugation (MNP no RPM), after one or two rounds of high speed RPM (MNP+RPM and MNP+2RPM respectively) and PEGylation (MNP+PEG).

The protocol of MNP synthesis by neutralization of dopamine hydrochloride solution with sodium hydroxide by auto-oxidation is illustrated in Figure 2. After overnight synthesis (12 hours) the MNP were purified through several centrifugation/redispersion cycles in deionized water to increase the uniformity of the MNP fraction. This process allowed us to obtain ∼0.37 mg/ml of MNP. Formation of melanin nanoparticles synthesized by neutralization of dopamine hydrochloride with sodium hydroxide, or with oxidation DOPA with KMnO_4_, has been verified by transmission electron microscopy (TEM) and NIR spectroscopy. TEM images showed the size of 48±12 nm and spherical shape of generated melanin-like nanoparticles before (Figure 3a) and after PEGylation (Figure 3b).

We developed a protocol that improves the conjugation process of PEG to MNP. A significant part of this protocol consists of the purification of MNP stock solution. Our modified protocol of PEGylation required a higher ratio of PEG/MNP and activation of process through potassium carbonate, K_2_CO_3_. The amount of mPEG thiol, which we used for binding to the MNPs, was 5 mg/mL for MNP solutions in concentration 150 μg/mL. PEG covered MNP does not have any morphological changes, which is demonstrated by comparison of Figure 3a and 3b. Pegylation of MNP was done for several important reasons. Pegylation is necessary for any *in vivo* application of MNP, because without it the MNPs would be recognized in the reticuloendothelial system (RES) or macrophage system and accumulate in liver and spleen, before reaching its destination or region of interest. PEG-MNP demonstrated strong stability (up to several weeks) in NaCl, PBS and fetal bovine serum solutions, where native MNP precipitated in several days.

UV-VIS-NIR absorption spectra presented for MNP after centrifugation with different speed followed by their PEGylation is shown in Figure 3c. Even though synthetic melanins are insoluble in water, we achieved excellent dispersion of MNPs in water using PEGylation. The surface of MNP can be effectively modified with thiol-terminated methoxy-poly (ethylene glycol), abbreviated as mPEG-SH with reaction between terminal thiol groups and the catechol/quinine groups of the polydopamine. Surface-modification of MNP was confirmed by characteristic of spectral peaks of PEG (alkyl C-H and C-O-C stretching) in literature [29]. MNP provide surface sites for amine- and/or thiol-functionalized molecules for a wide range of functionality for biomedical applications and imaging.

### 3.2 MNP toxicity and biocompatibility

Biocompatibility of pegylation was investigated through cell viability and proliferation using different cell lines. We used 2 different techniques to assess damage to the cell membrane, cell toxicity and cell viability. Combined data on LDH release, MTT conversion, and LDH_R/_Total LDH + LDH_R_ were also necessary for confirmation of the PEGylation protocol.

**Figure 4:**
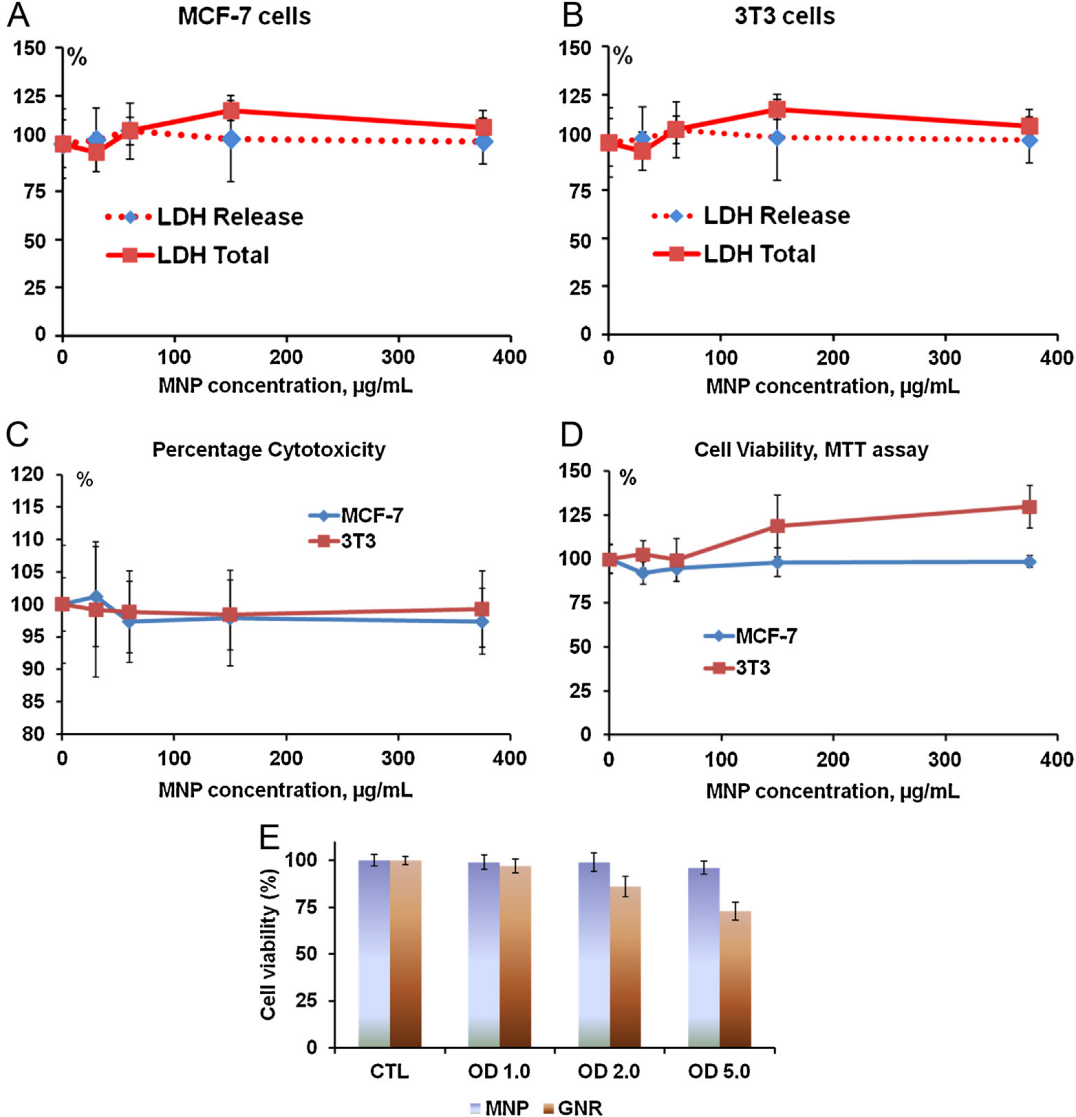
Dose dependence effects after 24 hours incubation MNP with MCF-7 and 3T3 cell lines on LDH release into medium and total quantity of LDH in cells after their membranes are destroyed with Triton X100 (LDH Total), each presented as mean ± SD from three independent samples per concentration. (c) Percent of cytotoxicity as a function of nanoparticle concentration after 24 hours of MNP incubation with MCF-7 and 3T3 cell lines; (d) Cell viability according to MTT assay as a function of nanoparticle concentration after 24 hours of MNP incubation with MCF-7 and 3T3 cell lines; (e) Viability of MCF-7 breast cancer cells measured by MTT assay as a function of optical density of MNP in water suspension and viability of SKBR3 breast cancer cells measured by the same assay as a function optical density of GNR suspension. Concentrations that correspond to OD=1, 2 and 5 are equal to 300, 600 and 1500 μg/mL for MNP and 25, 50 and 125 μg/mL for GNR, respectively.

We investigated the influence of concentrations of PEGylated MNP on the physiological status of cell cultures. MNP toxicity and cell viability was measured for two different human cell lines (MCF-7 and 3T3) using LDH (Figure 4a,b) and MTT (Figure 4c,d) assays kits (Roche) as a function of the dosage of surface-modified melanin nanoparticles with various concentrations (from 30 to 375 μg/mL for 24 h).

The Cytotoxicity Detection Kit measures cytotoxicity and cell lysis by detecting LDH activity released from cell, as a result of the damage to the plasma membrane after MNP administration. MTT is cleaved to formazan by the “succinate-tetrazolium reductase” system (EC 1.3.99.1), which belongs to the mitochondrial respiratory chain and is active only in viable cells or their metabolic activity [36]. We observed only minor differences in the measured parameters for PEG-MNP conjugates which indicated absence of toxic effects. More than that, for the 3T3 cell line we can show a trend to the stimulation of cell viability (Figure 4c). Our cell viability data for PEGylated MNPs matches well to that demonstrated previously for HeLa cells using WST-1 assay [29,33]. Figure 4e depicts viability of breast cancer cells after their incubation during 24 hours within substantially expanded range of nanoparticle concentrations and related optical density (OD=1, 2, 5). Cytotoxicity was studied by MTT assay for MNP solutions in MCF7 cell cultures, and toxicity of GNR was studied in SKBR3 cell culture. The same OD required different concentrations of MNP and GNR nanoparticles. OD 1, 2 and 5 correspond to concentration of 300, 600 and 1500 μg/mL for MNP and 25, 50 and 125 μg/mL for GNR [34], respectively. In addition, data published previously demonstrated similar toxicity of PEG-GNR, which was estimated by alternative methods of cell viability and cell membrane damage assays [35,44].

We demonstrated that PEG modified MNPs showed no cytotoxicity, no decrease in cell viability and no change in morphology of cell membranes for the concentration of MNP up to 1,500 μg/mL. Figure 4 summarize the results, which confirmed that PEGylated MNP have practically no toxicity with 3 different tests: LDH release, percentage of cytotoxicity (ratio LDH release and total LDH in cells) and cell viability (MTT). Enzymatic activity tests *in vitro* cell cultures cannot be performed with concentrations higher than 375 mg/mL, because background absorbance of MNP is too high for LDH assay to be measured with a spectrophotometer. However, motivated to determine the true concentration at which MNP toxicity begins, we performed an experiment using MTT assay with MNP concentrations up to C=3 mg/mL (OD=10). We observed the beginning of PEG-MNP cytotoxicity at MNP concentration of 1.5 mg/mL for MCF-7 cells (corresponding OD=5) and C=1.8 mg/mL for 3T3 cells (corresponding OD= 6). Thus, in one experiment, carefully performed, however without statistical significance, we demonstrated lack of MNP toxicity in concentrations about 4 times greater than concentrations measureable from LDH assay.

Since GNRs are widely used as optoacoustic contrast agents for preclinical research, it is interesting to compare toxicity of GNRs and MNPs. Let us consider MNPs having diameter of 50 nm and GNRs having length of 50 nm and width of 15 nm. The volume each GNR is about 8900 nm^3^. Mass of Au per NR is 8900 nm3 × 59 (Gold atoms/nm^3^) × 197/N_A_ (MW of Gold/Avogadro constant) ∼ 1.7 × 10^−10^ μg/GNR. As we found experimentally, water suspension of these type of GNRs with aspect ratio 3.34 has optical density of 1.0 in the concentration of 250 pM, which corresponds to ∼ 1.5 × 10^11^ GNR/mL or ∼ 25 μg GNR/mL [34,35].

For MNPs with OD=1 concentration is 0.3 mg/mL. There is ∼ 3 × 10^17^ nm^3^ per mL and the solution contains ∼ 5.2 × 10^12^ MNP/mL because average volume for studied MNP is 5.7×10^4^ nm^3^. Thereby the molar concentration value is ∼ 9×10^−9^ M (5.2 × 10^15^/N_A_). Therefore, concentration of MNP required to achieve optical density OD=1 is 12 times greater than that for GNRs.

Our previous studies of toxicity of GNR on different cell lines, such as breast cancer and non-cancer cells, leukemia cells, normal epithelial cells, and etc. [34,35,44] demonstrated that range of cyto-toxicity of PEGylated GNR has ranged between 50-75 μg GNR/mL (OD=2-3). In the present study we demonstrated using MTT assay that toxicity of MNPs begins to manifest itself at the average concentration of about 1.5 mg/mL (OD∼5). Therefore, toxicity of MNP is about 2 times lower than toxicity of GNR in suspensions of equal optical density.

### 3.3 Optoacoustic contrast of MNP in vitro and in vivo

**Figure 5:**
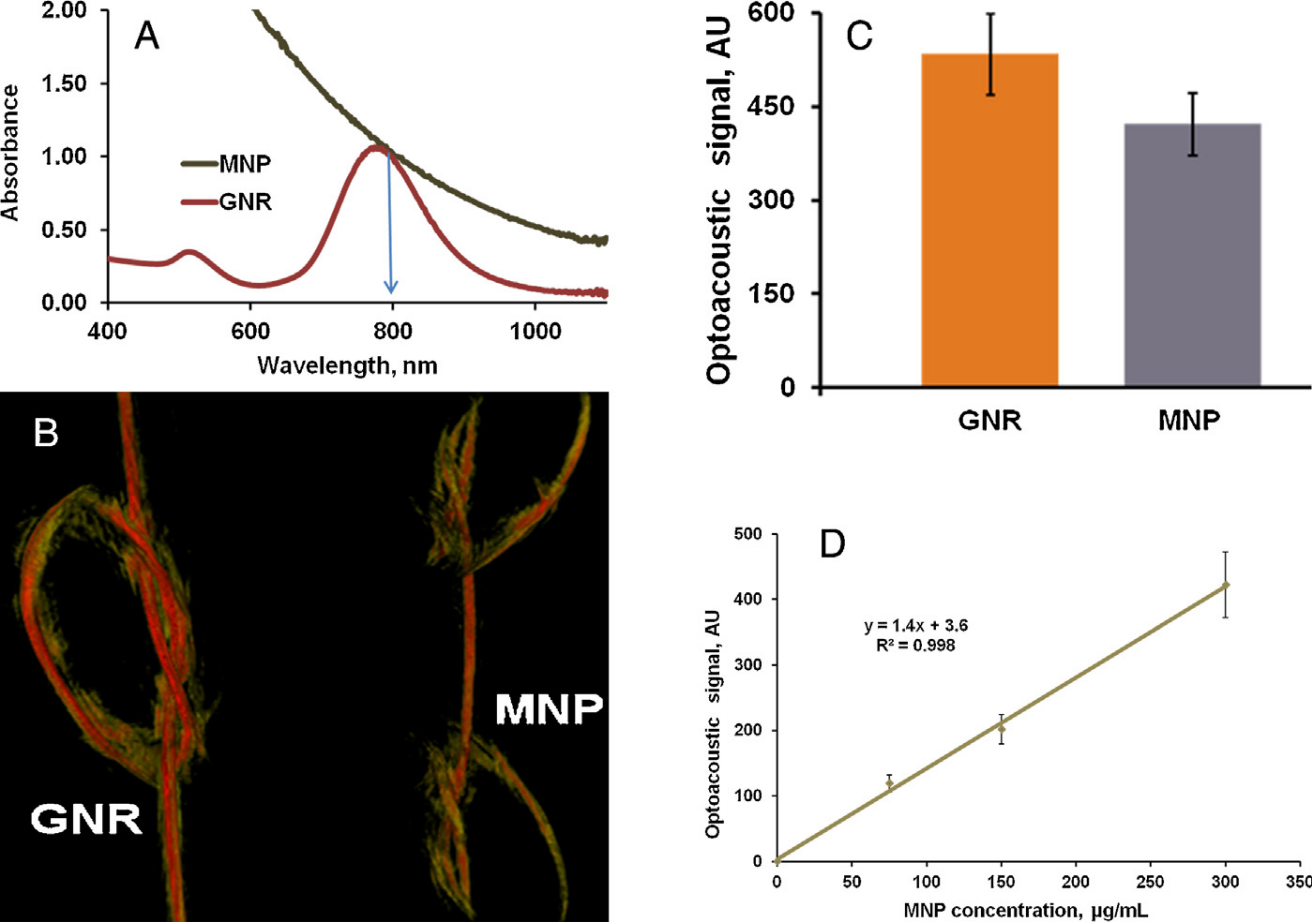
(a) Optical UV-VIS-NIR absorption spectra of MNP and GNR after equilibration at the wavelength of 800 nm shown with arrow. (b) OA image of two silicone tubes of internal diameter of 510 μm and wall thickness of 150 μm, GNR tube has a single knot (left) and MNP has two knots (right), both nanoparticle suspensions had equal optical density of 1.0. (c) The average optoacoustic brightness M ± SD of the tubes with GNR and MNP is presented as a histogram. (d) Optoacoustic image brightness of silicon tubes internal diameter of 510 μm and wall thickness of 150 μm filled with water suspension of MNP, showing linear dependence as a function of concentration. Points on the graph correspond to the optical density of 0.25, 0.5 and 1.0.

The optoacoustic contrast of synthesized MNP-PEG nanoparticles was tested in phantoms and two live mice. 3D optoacoustic tomography was applied to the visualization of tubes filled with water suspensions of MNPs and GNRs. Prior to the imaging, the solutions of MNP and GNR with equal optical density OD=1 at the wavelength 800 nm (see optical absorption spectra in Figure 5a) were prepared. Green arrow on Figure 5a indicates the wavelength 800 nm at which the optoacoustic imaging was performed.

We performed two OAT experiments to demonstrate the optoacoustic brightness of melanin nanoparticles relative to that of gold nanorods and measure the image brightness as a function of MNP concentration. OA image of a phantom containing two silicone tubes with internal diameter of 510 μm was acquired. Figure 5b depicts an image of GNR tube having a single knot (left) and MNP tube with two knots (right), both having equal optical density OD=1.0. Optoacoustic contrast presented in Figure 5b presents an average image brightness M ± SD over the central area evenly illuminated by the about equal optical fluence (knot area of the GNR tube and vertical area of the MNP tube). The imaging experiment was repeated three times. The average pixel brightness was found (considering experimental errors) approximately equal in both nanoparticle suspensions (Figure 5c). Because wall thickness was 150 μm we could estimate the image resolution (d < 0.3 mm) from the fact two tubes touching each other in the knot were well separated (see Figure 5b).

Further, the tubes were filled with two times and four times diluted concentrations of GNR and MNP. A linear correlation between the nanoparticle concentration and the optoacoustic image brightness was found (Figure 5d).

The signal to noise measurements performed in this experiment permitted an estimate of the system sensitivity. The minimum optoacoustic brightness that could be detected above the noise corresponded to the optical density of OD=0.1, which corresponds to Δμa=0.23/cm in the tube illuminated with combined effective optical fluence of about 2.5 mJ/cm^2^. Should one apply maximum safe level of the optical fluence permitted by ANSI (20 mJ/cm^2^) the minimum detectable change in the optical absorption coefficient will become ∼0.03/cm.

**Figure 6:**
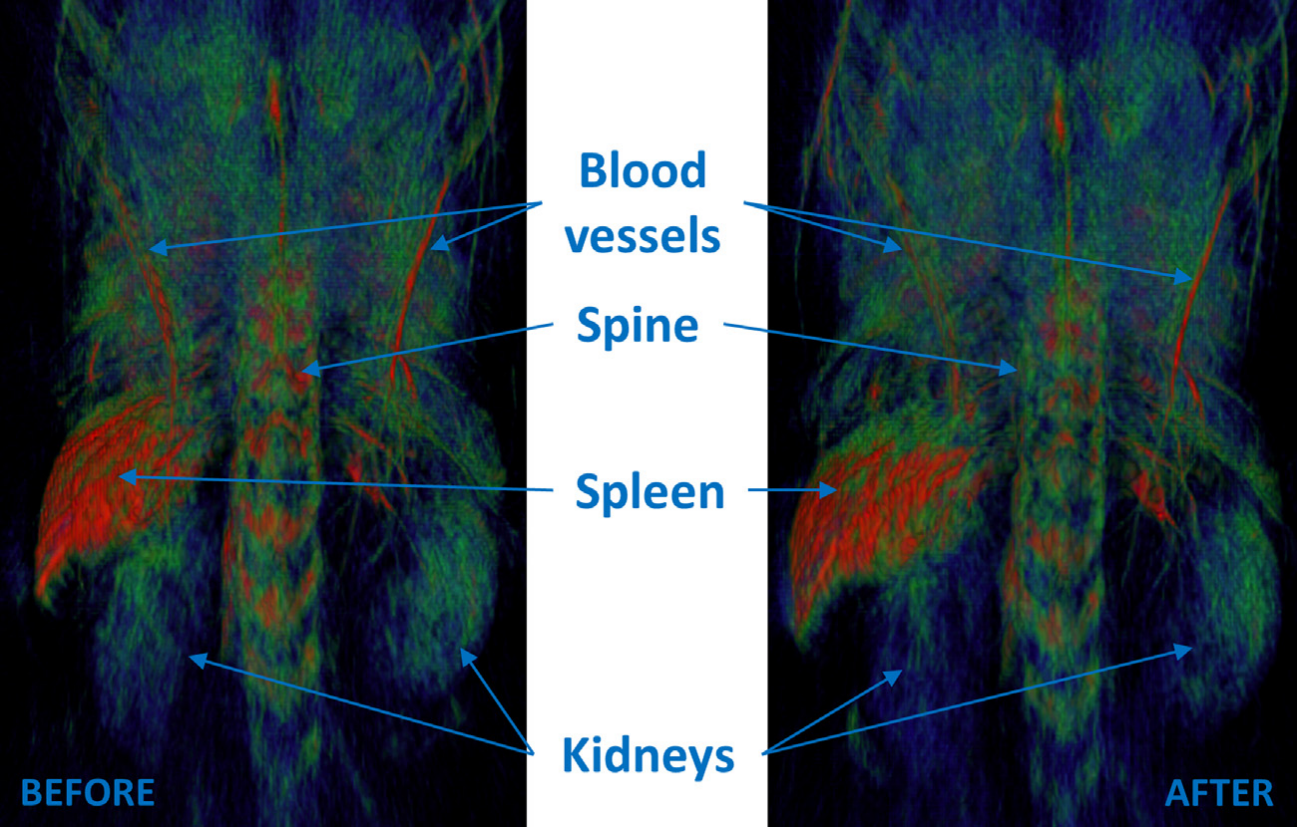
Dorso-ventral projection of the three-dimensional optoacoustic images of a live mouse before and 2 hours after intravenous injection of 0.2 mL MNPs with concentration C=30 mg/mL, which corresponds to about 200 μg/g body mass.

To investigate the biocompatibility of MNP, we administrated these nanoparticles in two live nude mice. PEGgylated MNP were injected intravenously in the tail vein of each mouse with a dose of 200 μg/g of body mass, or around 6 mg/per mouse (weight ∼29 g). Contrast of commonly seen organs such as spleen or spine, were not found significantly changed as compared with native contrast of hemoglobin (see Figure 6). This conclusion can be understood from the following consideration. The injected concentration of ∼30 mg MNP/mL which provides optical absorption of ∼100/cm at 800 nm. We injected 200 microliter volume, maximum volume permitted by our animal care protocol. Sometime injection though a tail vein is difficult, so injections in two live mice that resulted in similar optoacoustic images provided an assurance that we do not have a problem with administration of MNPs into the animal. The injected nanoparticles did not change the image contrast of the main features of the optoacoustic image: spleen, kidneys, vasculature and microvasculature of the spine, possibly even slightly reduced (see Figure 6), which could be explained by the fact that the optical absorption of male mouse blood with hematocrit of ∼38 at 800 nm is ∼8/cm, while 0.2 mL of MNP suspension injected into a 29 g mouse circulatory system with 2.6 mL of blood would result in 13 times dilution yielding the optical absorption of MNPs of 7.7/cm. We also attribute some slight decrease of brightness to a lower fluence used for the image acquisition from 1.2 mJ/cm^2^ in the image acquired before injection to 1.1 mJ/cm^2^ applied for post injection imaging. We need to note, however, that the main purpose of our *in vivo* experiment was to demonstrate that MNPs are non-toxic neither acutely nor long term in concentrations that after dilution by blood in the circulatory system of a mouse will yield optical absorption close to that of blood. While we did not target any specific region of interest (our nanoparticles did not have a targeting agent), we aimed at understanding of possible biodistribution and accumulation of MNPs in the mouse body. To our satisfaction, from analysis of the optoacoustic image contrast we determined that MNPs did not accumulate in organs such as kidneys, spleen and liver two hours post injection, indicating that they are distributed in the circulatory system, which should enable successful targeting of tumors or other tissues of medical interest. Mice survived MNP injection for over four weeks (longer period of keeping the animals was not permitted in our animal care protocol). Considering that toxicity of MNPs is about two times lower than that of GNRs per unit of the optical absorption, one can potentially achieve a greater enhancement of the optoacoustic contrast while imaging live animals with MNPs though local accumulation in the region of interest. Future more comprehensive studies will determine the toxicity and mortality effects of MNPs in longitudinal studies, which require a large number of mice for statistical inference.

## 4 Conclusion

We synthesized and tested a novel efficient melanin nanoparticle-based contrast agent for optoacoustic imaging. Using a size control synthesis melanin nanoparticles with diameter of 50 nm were produced. A specially designed PEGylation process yielded nontoxic PEG-MNP conjugates that remain stable for at least 8 weeks in biological media, such as PBS and serum solutions and no noticeable changes in their optical properties were observed. Using tubing phantoms we showed that optoacoustic efficiency of MNPs is similar to that of GNRs. MNP-PEG was found non-toxic and biocompatible with human MCF-7 and 3T3 cells with toxicity level at least 20 times lower than that of GNR-PEG with the same concentration. Considering that the concentration (mass of nanoparticles per mL) needed to achieve equal OD is 12 times greater for MNP than for GNR, the cyto-toxicity of MNPs is about 2 times lower than that of GNRs if measured in units of [μg/mL]/OD. Therefore, one can potentially achieve a greater enhancement of the optoacoustic contrast while imaging live animals with MNPs. Injection of 0.2 mL of PEG-MNPs with about maximum achievable concentration of 30 mg/mL in a live mouse resulted in no life threatening toxicity and long lasting circulation without noticeable accumulation in vital organs within two hours. Given the fact that MNPs are biodegradable and GNR are not, further studies of MNPs as optoacoustic contrast agent are warranted, especially having in mind clinical applications.
